# Viral Etiology of acute respiratory tract infections in hospitalized children and adults in Shandong Province, China

**DOI:** 10.1186/s12985-015-0388-z

**Published:** 2015-10-14

**Authors:** Ti Liu, Zhong Li, Shengyang Zhang, Shaoxia Song, Wu Julong, Yi Lin, Nongjian Guo, Chunyan Xing, Aiqiang Xu, Zhenqiang Bi, Xianjun Wang

**Affiliations:** Shandong Center for Disease Control and Prevention, Shandong Provincial Key Laboratory of Infectious Diseases Control and Prevention, Shandong University Institute for Prevention Medicine, Jinan, 250014 Shandong China; Jinan Central Hospital Affiliated to Shandong University, Jinan, 250014 Shandong China

**Keywords:** Hospitalized patients, Acute respiratory virus infection, Etiology, Epidemiology

## Abstract

**Background:**

The dominant viral etiologies responsible for acute respiratory infections (ARIs) are poorly understood, particularly among hospitalized patients. Improved etiological insight is needed to improve clinical management and prevention of ARIs.

**Methods:**

Clinical and demographic information and throat swabs were collected from 607 patients from 2011 to 2013 in Shandong Province, China. Multiplex RT-PCR (SeeplexTM RV detection, Seegene) was performed to detected 12 respiratory viral pathogens.

**Results:**

A total of 607 hospitalized patients were enrolled from 2011 to 2013. Viruses were identified in 35.75 % (217/607) of cases, including 78 influenza virus A and B (IVA and IVB), 47 para-influenza viruses (PIVs), 41 respiratory syncytial virus (RSV) and 38 adenovirus (ADV). For the children under 15 year old, the common detected viruses were influenza viruses, RSV, PIVS and ADV, while the principal respiratory viruses were human coronaviruses (HCoV), PIVs, influenza viruses for the old adults. Co-infections with multiple viruses were detected in 15.67 % of patients. Children under 5 years were more likely to have one or more detectable virus associated with their ARI. The peak of ARI caused by the respiratory viruses occurred in winter.

**Conclusion:**

This study demonstrated respiratory viruses were the major cause of hospitalized ARI patients in Shandong Province, influenza virus was the most common detected, RSV was the highest incidence among the young children (≤5 years). These findings also gave a better understand of virus distribution among different age and seasons, which help to consider potential therapeutic approaches and develop effective prevention strategies for respiratory virus infection.

## Background

The World Health Organization (WHO) estimates that acute respiratory infections (ARIs) cause nearly four million deaths per year, a rate of more than 60 deaths/100,000 population [1]. Rates are even higher in developing countries, where pneumonia is responsible for an estimated 10–25 % of all deaths among children under 5 years of age [2]. A lot of pathogens can cause ARIs, and viruses have been considered as the main pathogens in people [3]. The major viral agents of ARIs include influenza viruses A and B (IAV,IBV), respiratory syncytial virus (RSV), para-influenza viruses (PIVs), adenovirus (ADV) and human rhinovirus (HRV). In the past decade, several new viruses associated with ARIs such as human metapneumovirus (hMPV) and human coronaviruses (HCoV) [4] have been discovered in human respiratory tract specimens.

Currently, there are no useful vaccines for preventing the infection of the respiratory viruses. A clear knowledge of the viral etiology of hospitalized ARIs in different age groups is critical to the successful implementation of the prevention, control and treatment strategies. Because of the geographical or climatic differences, or socioeconomic factors, the epidemiological presentation of viral etiology varied among different study population in different countries or regions [5–12].

China is a large country with different climate characteristics among different regions. A better understanding of the viral etiology of hospitalized ARIs in different regions plays a predominant role for the local prevention, control and treatment of ARIs. Although several studies on the epidemiology of ARIs have recently reported in Beijing, Shenzhen, Hong Kong, Gansu province and China, the epidemic profiles of viruses in ARIs are different because of different enrolled criteria, geographical and climatic factors [8–13].

Shandong locates in eastern China and lies near to Southern Korean and Japanese with 97 million populations, which has a large transient population from different provinces and counties. The mixing of transient population may increase the transmission of respiratory viruses. Feng et al. [10] reported the viral etiology of hospitalized acute lower respiratory infection patients in 22 provinces of China, including Shandong province, but the findings didn’t include the data in this study. At the same time, HRV as the major infection pathogen was detected in the hospitalized ARIs, but Feng’s result didn’t describe HRV prevalence. Thus, our aim was to investigate the frequency and type of twelve common respiratory viral infections in hospitalized ARIs among children and adults in Shandong Province from 2011 to 2013.

## Results

### Patient characteristics and clinical diagnosis

During January 2011-December 2013, 607 specimens were collected from patients with hospitalized ARIs in the present study. The patients were 348 (57.33 %) males and 259(42.67 %) females (sex ratio 1.34; Table 1) (*x*^*2*^ = 0.06, *P* > 0.5). Among the 607 ARI patients, 40.03 % were children aged <5 years and 21.42 % were elderly aged ≥ 60 years, with a median age of 6 years (range, 1 months to 99 years) (Table 1). A temperature ≥ 38 °C was documented in 96.54 % of ARI cases at the time of physical examination. The most common symptoms observed were cough (78.25 %), followed by sore throat (47.78 %). The respiratory symptoms are described in Table 1.

**Table 1:** Summary of associations between clinical characteristic and viral infectious

Characteristic	ARI (%)(*N* = 607)	Infected sample(*N* = 217)
sex		
Male	348(57.33 %)	123(56.68 %)
Female	259(42.67 %)	94(43.32 %)
Age		
≤2 years	125(20.59 %)	52(23.96 %)
3 ~ 4	118(19.44 %)	57(26.27 %)
5 ~ 15	124(20.43 %)	50(23.05 %)
16 ~ 59	110(18.12 %)	29(13.36 %)
≥60 years	130(21.42 %)	29(13.36 %)
Chronic disease		
chronic lung disease	57(9.39 %)	14(6.45 %)
coronary artery heart disease	123(20.26 %)	32(14.75 %)
Metabolic	48(7.91 %)	15(6.91 %)
incubate the influenza vaccine last year	31(5.11 %)	18(8.29 %)
Clinical Symptom		
fever(<38)	21(3.46 %)	8(3.69 %)
fever (≥38)	586(96.54 %)	209(96.31 %)
sore throat	290(47.78 %)	88(40.55 %)
Cough	475(78.25 %)	175(80.65 %)
difficulty breathing	173(28.50 %)	55(25.34 %)
lung auscultation	357(58.81 %)	119(54.84 %)
abnormal X-ray	270(44.48 %)	83(38.25 %)

### Viral etiologies

The NTS were analyzed by multiple RT-PCR, 217 (35.75 %) specimens carried at least one virus, comprising 183 single-infection cases (84.33 %), and 34 co-infection cases (15.67 %)(Table 2). The most frequently detected virus was influenza virus (30.47 %, 78/256), followed by PIVs (18.36 %, 47/256), RSV (16.02 %, 41/256) and ADV (14.84 %, 38/256) (Table 2).

**Table 2:** Viral etiologies identified (2011–2013)

Infections	Single infection (%)	Co-infection (%)	Total (%)
	*N* = 183(30.15)	*N* = 34(5.60)	*N* = 217(35.75)
IAV	39(21.32)	9(12.33)	48(18.75)
IBV	17(9.29)	13(17.81)	30(11.72)
ADV	29(15.85)	9(12.33)	38(14.84)
RSVA	15(8.20)	14(19.18)	29(11.33)
RSVB	11(6.01)	1(1.37)	12(4.69)
PIV-1	8(4.37)	3(4.11)	11(4.30)
PIV-2	15(8.20)	2(2.74)	17(6.64)
PIV-3	14(7.65)	5(6.85)	19(7.42)
HCoV(229E/NL63)	7(3.83)	3(4.11)	10(3.91)
HCoV(OC43/HKU1)	8(4.37)	4(5.48)	12(4.69)
HRV	20(10.93)	6(8.22)	26(10.16)
HMPV	0(0)	4(5.48)	4(1.56)
Total	183	73 (34 cases)	256

Among the 34 ARI cases with co-infection, two viruses were identified in 29 patients (85.29 %) and three viruses were detected in 5 patients (14.71 %) (Table 2). Among the co-infected specimens, the most common virus was RSVA (19.18 %), followed by IBV (17.81 %), AdV and IAV (12.33 %) (Table 2).

The total positive rates in the year of 2011, 2012 and 2013 were 41.86, 42.69 and 29.32 %, respectively. It was statistically lower in 2013 than in the other 2 years (*x*^*2*^ = 11.22, *P* < 0.01), but no difference was observed between 2011 and 2012 (Table 3).

**Table 3:** Association between years and respiratory virus infection (2011–2013)

Infections	Years		
	2011	2012	2013
	(*n* = 129) (%)	(*n* = 171) (%)	(*n* = 307) (%)
IAV	19(14.73)	10(5.85)	19(6.19)
IBV	4(3.10)	25(14.62)	1(0.33)
ADV	18(13.95)	4(2.34)	16(5.21)
RSVA	4(3.10)	12(7.02)	13(4.23)
RSVB	1(0.78)	9(5.26)	2(0.65)
PIV-1	2(1.55)	3(1.75)	6(1.95)
PIV-2	4(3.10)	4(2.34)	9(2.93)
PIV-3	4(3.10)	4(2.34)	11(3.58)
HCoV(229E/NL63)	3(2.33)	2(1.17)	5(1.63)
HCoV(OC43/HKU1	3(2.33)	6(3.51)	3(0.98)
HRV	2(1.55)	11(6.43)	13(4.23)
HMPV	2(1.55)	2(1.17)	0(0)
single infectious	42(32.56)	58(33.92)	83(27.04)
co-infectious	12(9.30)	15(8.77)	7(2.28)
positive cases	54(41.86)	73(42.69)	90(29.32)

### Age and gender distribution

All of the ARI patients were grouped into five age groups with different positive rate of viral infections (Table 1 and Table 4). The overall positive rate was double in young Children (<5 years) than that in old adults (>60 years) (44.85 VS 22.31 %). All of the detected viruses predominated in children (≤15 years old), except for HCoV which dominated in the adults (>15 years old). The predominant viruses among different age groups were different. Of young children (≤2 years), RSV was the most prevalent virus followed by PIVs, ADV and IVA. Of Children aged 3 ~ 4 years, IVA and RSV had the highest incidence, followed by IVB and HRV. Of persons aged 5 ~ 15 years, ADV was the most common pathogen, followed by IVA and PIVs. For the person aged ≥60 years, the incidence of PIVs and HCoV were the highest, followed by RSVB(Table 4). There was no significant difference in viral infection between male (35.34 %, 123/348) and female. (36.29 %, 94/259) (*p* ≥ 0.05).

**Table 4:** Association between age groups and respiratory virus infection (2011–2013)

Infections	Age group				
	≤2 year	3 ~ 4	5 ~ 15	16 ~ 59	≥60
	(*n* = 125) (%)	(*n* = 118) (%)	(*n* = 124) (%)	(*n* = 110) (%)	(*n* = 130) (%)
IAV	9(7.20)	13(11.02)	10(8.06)	13(11.82)	3(2.31)
IBV	4(3.20)	12(10.17)	9(7.26)	2(1.82)	3(2.31)
ADV	10(8.00)	7(5.93)	15(12.10)	6(5.45)	0(0)
RSVA	13(10.40)	7(5.93)	4(3.23)	1(0.91)	4(3.08)
RSVB	6(4.80)	6(5.08)	0(0)	0(0)	0(0)
PIV-1	5(4.00)	3(2.54)	2(1.61)	1(0.91)	0(0)
PIV-2	1(0.80)	3(2.54)	6(4.84)	4(3.64)	3(2.31)
PIV-3	8(6.40)	3(2.54)	2(1.61)	0(0)	6(4.62)
HCoV(229E/NL63)	1(0.80)	3(2.54)	1(0.81)	1(0.91)	4(3.08)
HCoV(OC43/HKU1	4(3.20)	0(0)	1(0.81)	2(1.82)	5(3.85)
HRV	3(2.40)	10(8.47)	7(5.65)	3(2.73)	3(2.31)
HMPV	2(1.60)	1(0.85)	0(0)	0(0)	1(0.77)
single infectious	40(32.00)	47(39.83)	44(35.48)	25(22.73)	27(20.77)
co-infectious	12(9.60)	10(8.47)	6(4.84)	4(3.64)	2(1.54)
positive cases	52(41.60)	57(48.31)	50(40.32)	29(26.36)	29(22.31)

### Seasonal distribution

**Fig. 1:**
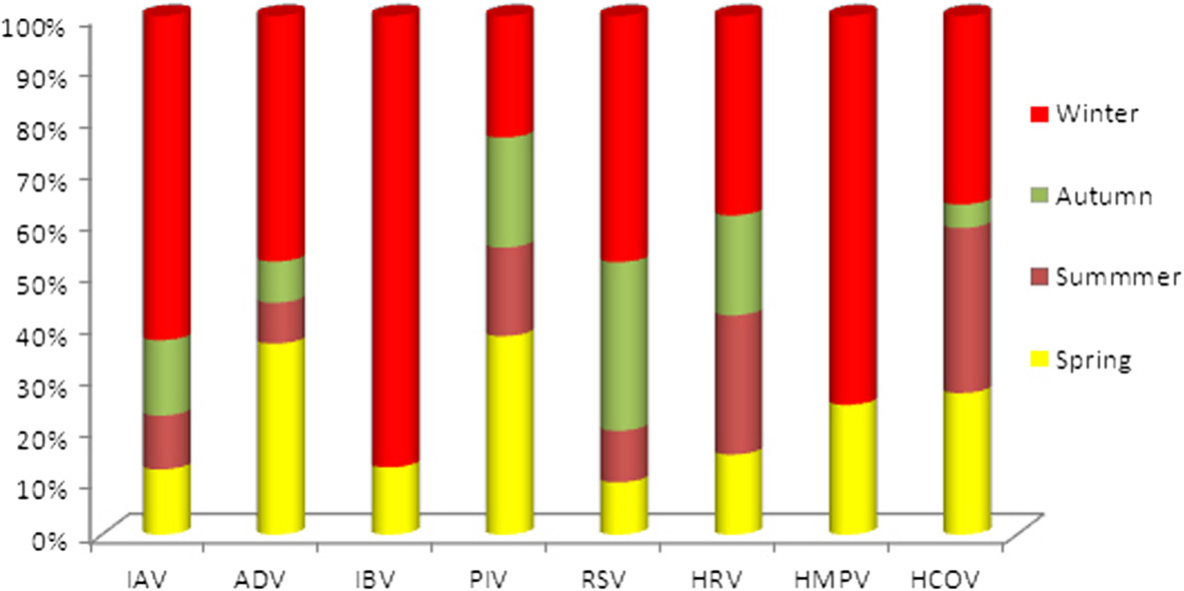
Seasonal distributions of individual viruses. Respiratory viruses were detected over four seasons with the prevalence expressed as a percentage of the total

The virus detection rate was not distributed equally during different seasons in Shandong Province (Fig. 1). The total positive rate was the highest in the winter (49.22 %), followed by spring (22.27 %), autumn (15.23 %) and summer (13.28 %). For the every virus, IAV and IBV were mostly detected in winter, while RSV was detected primarily during the autumn and winter. HRV and PIVs were detected in all seasons, while ADV was detected primarily during the spring and summer. Other viral pathogens appeared sporadically during the year.

#### Clinical characteristics of patients with viral infection

The clinical symptoms and diagnosis of the patients with viral infection are shown in Table 5. Fever and cough compares the majority of clinical symptom. Pneumonia was the most common diagnosis for RSV, PIVs, HRV, IVA, HCoV and ADV infection, while IBV had the highest rate of bronchitis than the other viral infection.

**Table 5:** Clinical symptoms and diagnosis results from infected patients

virus	number	fever	cough	throat	difficulty breathing	lung auscultation	abnormal X ray	diagnose result	
								pneumonia	bronchitis
RSV	41	41(100)	32(78.05)	18(43.90)	6(14.63)	25(60.98)	15(36.59)	31(75.61)	4(9.75)
IVA	48	47(97.92)	38(79.17)	20(41.67)	13(27.08)	35(72.92)	21(43.75)	30(62.5)	8(16.67)
HRV	26	26(100.00)	19(73.08)	13(50.00)	9(34.62)	8(30.77)	7(26.92)	18(69.23)	3(11.54)
PIV	47	43(91.49)	36(76.60)	19(40.43)	15(31.91)	27(57.45)	22(46.81)	34(72.34)	4(8.51)
ADV	38	34(89.47)	28(73.68)	16(42.11)	8(21.05)	16(42.11)	1(2.63)	22(57.89)	7(18.42)
IVB	30	30(100.00)	26(86.67)	8(26.67)	5(16.67)	10(33.33)	7(23.33)	13(43.33)	13(43.33)
HCoV	22	22(100.00)	21(95.45)	10(45.45)	5(22.73)	15(68.18)	10(45.45)	13(59.09)	5(22.73)

## Discussion

Respiratory viruses causing acute respiratory infection are a significant source of morbidity and mortality, especially in children under 5 years. There were some research reports about the epidemiology and etiology of respiratory viruses among the children throughout the world, but study of hospitalized ARIs in children and adults simultaneous is more limited. Due to the different age groups, climate and other factors, the infection incidence, seasonality, co-infection rate and clinical profiles of respiratory virus in hospitalized ARIs are different. In this study, a total of 607 hospitalized ARI cases were enrolled from 2011 to 2013, and 35.75 % were positive for at lease one virus, which was consistent with the previous study in China (36.6 %) [10]. The finding also illustrated that the virus positive rate was double in children less than 5 years than that in adults. At the same time, children less than 5 years old accounted for most cases of ARIs (44.15 %) and 45.52 % of them had a documented viral infection (Table 1), a similar incidence rate has been obtained in Shenzhen, Hong Kong, Rome and Milan [13–16], but it was different from other studies [17, 18]. For the old adult (≥60 years), viruses were detected in 22.31 % (29/130) of samples, which was lower than that of the study reported by Ren L [12] and Raquel Cirlene da Silva [19].

For all of the hospitalized ARIs, the principal pathogens were IVA, PIVs (PIV1-3), RSV, ADV and IBV. For the young children (≤5 year), the most prevalent viruses were RSV, PIVs, IVA, ADV and IVB, while the principal respiratory viruses were HCoV, PIVs, RSV in the old adult groups. The epidemic characteristic differed among the age groups and seasonality.

Influenza viruses were the most frequently detected respiratory viruses in all hospitalized ARIs, accounting for 12.85 % (78/607), and IVA (7.91 %, 48/607) was more prevalent than IVB (4.94 %, 30/607). For the young children less than 5 years, the positive rate was three times than that in old adults (15.63 % VS 4.62 %). The influenza virus A(H1N1) pdm09 and dynamic change of influenza viruses could explain this shift. As an emergence infectious disease, people could be vulnerable to infection with influenza virus A (H1N1) pdm09 because of low antibody in the second wave in 2010–2011 surveillance season [20]. The influenza surveillance data also verified that influenza A (H1N1) pdm09 was the main strain in Shandong in 2011 and 2013 [21]. After that in 2011, IVB became the predominant strain in 2012 due to the limited immunological cross-reactivity between influenza subtypes which lead to the increase of the infection population [20, 21]. Our study also highlighted a clear seasonal distribution of influenza viruses which were active in winter and IVA was detected in the four seasons.

PIV is a major cause of respiratory tract illness in infants and young children worldwide [22]. For the children under 5 years old, they were infected by at lease one PIV, and re-infected throughout life because of incomplete immunity [23]. PIV was the second pathogen detected in all hospitalized ARIs (positive rate of 7.41 %) and were found to be prevalent in almost all age groups. These data were consistent with the positive rate observed in Hong Kong and Rome. Compared with the 5.71 % positive rate in adults, our study also recorded a 10.07 % prevalence of PIVs infections under 5 years old with the predominate type being PIV-3, which was similar with the reports [24, 25]. At the same time, different from the prevalence of influenza virus in winter, PIV was active in all the seasons.

Our findings illustrated that RSV was the most pathogen of respiratory tract infection in children less than 5 years, accounting for 13.17 % (32/243), which were consistent with the study from Asia and China [26–28]. Our surveillance data also indicated that RSV-positive ARIs occurred during autumn and winter, which was in agreement with report from Jinzhou in China, Japan and the Unite States [28–30], but different from Hong Kong [13]. The seasonal characteristics of RSV infection may be related to a region’s climate and demographic factors. All of these would be important for local pediatricians to use antibiotics cautiously when children are hospitalized with ARIs.

It was known that ADV accounted for 5–10 % of lower respiratory tract infections with the highest rate occurring in young children [31, 32]. Our results also showed that the total positive rate was 6.26 %, with 60.53 % (23/38) patients were children under 5 years old. Most ADV (29 of 38) were detected as single infection, which agreed with the report of Huang et al. [9].

Human cororavirus (HCoV), an important pathogen in adults [33], was detected in 3.62 % of patients, which was significantly lower than the Xiaoyan Yu’s research [11], but higher than the previous report [34]. To pursue the reason, the detection methods or the changed prevalent rules every 2–3 years maybe the main reason [35]. Our surveillance data showed that the virus positive rate in the adults was two times than that in children. Consistent with previous study [36, 37], only four patients (0.66 %) were infected with hMPV, including three children between 9 months to 5 years.

The result described the different positive rate from 2011–2013, the probably reason may be the dynamic distribution of respiratory viruses and the collected sample amount in different months. In 2012, IBV had the highest incidence (14.62 %), accounting for 34.25 % of all positive samples, followed by RSV, HRV and PIVs. In 2011, IAV was the most common virus in 2011, followed by ADV and PIVs, while IVA was the predominant pathogen in 2013, followed by PIVs, ADV and RSV. At the same time, the rate of the sample amount in 2013 (48.21 %) was higher than the other 2 years (39.18 and 19.38 %) in the non-epidemic season of respiratory virus infection (May-October) which lead to the decrease of positive rate in 2013.

In our study, respiratory viral infections have clear seasonal variations with most cases occurring in the winter. Possible explanations for this include seasonal variations in host immune response to infection [33], climatic factors such as lower temperature and low relative humidity which increase viral survival in the environment [34].

Patient with respiratory viral infection usually develop clinical symptoms, including fever and cough, which further develop pneumonia and bronchitis. We found that RSV (75.61 %) and PIVs (72.34 %) infection were more likely to be associated with pneumonia than were ADV (57.89 %) and HCoV (59.09 %), while IVB shared the same chance to develop pneumonia and bronchitis (43.33 %).

## Conclusions

In conclusion, the results of our study give us a better understanding of the viral etiology of hospitalized ARIs in Shandong Province, China, which is useful for the guidance of pediatric clinical practice including the correct application of antibiotics as well as public health policy. Moreover, the use of multiple RT-PCR permits a rapid differential diagnosis of hospitalized ARIs potentially enabling rapid detection and response to outbreak.

## Methods

### Patients and specimens

From Jan, 2011, to December, 2013, the nasal and throat swabs (NTS) from Jinan Central Hospital Affiliated to Shandong University were collected from hospitalized ARIs patients. Selection criteria included having one or more respiratory symptoms, including cough, sore throat, combined with a body temperature above 37.5 °C. The other information including symptoms, clinical diagnosis and demographic characteristic were recorded in case report forms. Clinical information of patients with virus infection was reviewed retrospectively from the records. NTS were kept in viral transport medium and stored at −70 °C prior to analysis.

This study was approved by the Ethics Committee of Shandong Center for disease control and prevention and all patients signed a “Written Informed Consent”.

### Molecular detection of respiratory viruses

The viral nucleic acid was directly extracted from the clinical specimens by using a QIAamp mini viral RNA extraction Kit (Qiagen, German). The cDNAs were synthesized by PrimeScript TM 1st strand cDNA Synthesis Kit (Takara # 6110), all samples were tested by multiple RT-PCR screening according to Seeplex® RV12 ACE Detection manufacture (Seegene Cat No. RV6C00Y). The detected respiratory viruses included IVA, IVB, ADV, RSVA, RSVB, PIV1–3, hMPV, HCoV-229E/NL63, OC43/HKU1, and HRV.

### Statistical analyses

Statistical analyses were conducted using SPSS 17. Descriptive statistics were used to characterize the median age and the infection rates. The chi-squared test was used to compare the infection rates for respiratory viruses among different age groups and different clinic characters. *P*-value <0.05 was considered to be statistically significant.

## Abbreviations

WHO: World Health Organization
ARIs: Acute respiratory infections (ARIs)
IAV: Influenza virus A
IBV: Influenza virus B
RSV: Respiratory syncytial virus
PIV: Para-influenza viruses
ADV: Adenovirus
HRV: Human rhinovirus
HMPV: Human metapneumovirus
HCoV: Human coronaviruses
